# A Reservoir Species for the Emerging Amphibian Pathogen *Batrachochytrium dendrobatidis* Thrives in a Landscape Decimated by Disease

**DOI:** 10.1371/journal.pone.0033567

**Published:** 2012-03-12

**Authors:** Natalie M. M. Reeder, Allan P. Pessier, Vance T. Vredenburg

**Affiliations:** 1 Department of Biology, San Francisco State University, San Francisco, California, United States of America; 2 Wildlife Disease Laboratories, Institute for Conservation Research, San Diego Zoo Global, San Diego, California, United States of America; Duke University Medical Center, United States of America

## Abstract

Chytridiomycosis, a disease caused by the fungal pathogen *Batrachochytrium dendrobatidis* (*Bd*), is driving amphibian declines and extinctions in protected areas globally. The introduction of invasive reservoir species has been implicated in the spread of *Bd* but does not explain the appearance of the pathogen in remote protected areas. In the high elevation (>1500 m) Sierra Nevada of California, the native Pacific chorus frog, *Pseudacris regilla*, appears unaffected by chytridiomycosis while sympatric species experience catastrophic declines. We investigated whether *P. regilla* is a reservoir of *Bd* by comparing habitat occupancy before and after a major *Bd* outbreak and measuring infection in *P. regilla* in the field, monitoring susceptibility of *P. regilla* to *Bd* in the laboratory, examining tissues with histology to determine patterns of infection, and using an innovative soak technique to determine individual output of *Bd* zoospores in water. *Pseudacris regilla* persists at 100% of sites where a sympatric species has been extirpated from 72% in synchrony with a wave of *Bd*. In the laboratory, *P. regilla* carried loads of *Bd* as much as an order of magnitude higher than loads found lethal to sympatric species. Histology shows heavy *Bd* infection in patchy areas next to normal skin, a possible mechanism for tolerance. The soak technique was 77.8% effective at detecting *Bd* in water and showed an average output of 68 zoospores per minute per individual. The results of this study suggest *P. regilla* should act as a *Bd* reservoir and provide evidence of a tolerance mechanism in a reservoir species.

## Introduction

With forty-three percent of species in decline, amphibians are the hardest hit group in what is being called a possible sixth mass extinction [1]. Habitat loss [2], introduced predators [3], climate change [4], and disease [5] have all been implicated as synergistic causes. Chytridiomycosis is the disease resulting from infection with an aquatic chytridiomycete fungus, *Batrachochytrium dendrobatidis (Bd)* [5], [6], and is responsible for the declines or extinctions of about 200 of the 6,674 known amphibian species [7]. Most alarmingly, many declines are being documented in pristine and protected areas and it is unknown how this aquatic fungus spreads across land [8]. In the decade since *Bd* was described it has shown varying outcomes of infection among different species of amphibians ranging from tolerant reservoir species that are infected with *Bd* but do not show symptoms of chytridiomycosis to susceptible species that rapidly develop lethal chytridiomycosis. Reservoir species are of special concern as a potential mechanism of spread of *Bd* to new locations and a source of *Bd* in the environment.

The best-studied examples of *Bd*-tolerant carrier species are the African clawed frog, *Xenopus laevis*, and the American bullfrog, *Rana catesbeiana*. Both species survive with asymptomatic, low-level *Bd* infections [9], [10], [11] and both have been implicated in the global spread of *Bd* [12], [13]. The human-mediated dispersal of the highly aquatic *R. catesbeiana* and *X. laevis* explains how *Bd* was introduced across oceans, but cannot explain the introduction of *Bd* to remote areas. In eastern Australia a widespread native species (*Litoria wilcoxi*) survives with high levels of *Bd* prevalence and intensity and has been implicated as a reservoir species [14], [15]. In California, the Pacific chorus frog, *Pseudacris regilla*, has been identified as a possible reservoir species based on ecological restraints preventing other species from spreading *Bd* [16].

In the high elevation (>1500 m) Sierra Nevada of California chytridiomycosis-related declines of the mountain yellow-legged frog, a species complex of *Rana muscosa* and *Rana sierrae* [17], have been well documented. *Rana muscosa/sierrae* was once the most abundant vertebrate in the high Sierra before introduction of predatory fish and outbreaks of chytridiomycosis reduced the species to approximately five percent of its historic range [17]. Healthy populations of *P. regilla* found throughout the present and historic range of *R. muscosa/sierrae* suggest *P. regilla* may not be susceptible to chytridiomycosis. As a semi-aquatic, wide-ranging species, if *P. regilla* is a reservoir of *Bd* it may play an important role in the dynamics of chytridiomycosis-mediated declines of *R. muscosa/sierrae* in the Sierra Nevada. In this study, we test the hypothesis that *P. regilla* may play an important role in the spread of *Bd* by monitoring infected individuals in the lab. Lab studies are supported with field data gathered to detect changes in *P. regilla* populations in a basin with well-documented extirpation of *R. muscosa* in synchrony with a wave of *Bd*. We investigate potential mechanisms of tolerance with histological examination of infected skin. In addition, we use a soak technique to determine the number of zoospores an individual *P. regilla* adds on average to the zoospore pool in the Sierra Nevada with implications for survival of *R. muscosa/sierrae* populations.

## Results

### Infection prevalence and outcome in the field

**Figure 1:**
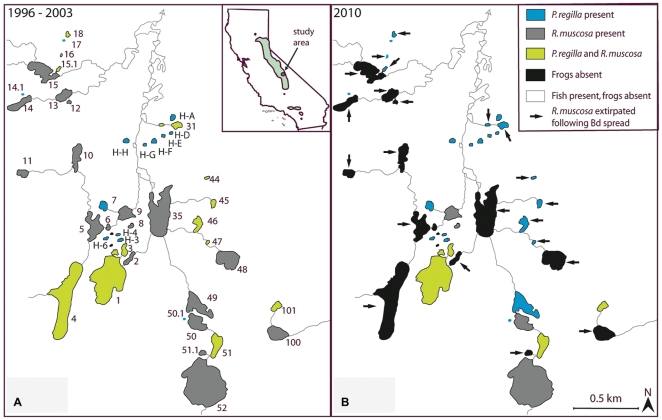
Habitat occupancy before and after disease spread. Range of *P. regilla* and *R. muscosa* in 60 Lake Basin (a) before and (b) after the spread of *Bd* through the basin.

In 60 Lakes Basin in Kings Canyon National Park, *P. regilla* remained present in 2010 at the 26 water bodies in which it was found in 2003 and colonized one additional site. Of 12 skin swabs taken in 2009 from *P. regilla*, 8 were positive for *Bd* as measured by PCR for an infection prevalence of 67%. *Rana muscosa* was present in 31 water bodies in 2003, but was extinct from all but 9 water bodies in 2010. *Rana muscosa* population extinctions followed the spread of *Bd* across the basin from North to South, but *P. regilla* population presence was unaffected by disease presence (Fig. 1).

### Laboratory infection monitoring

**Figure 2:**
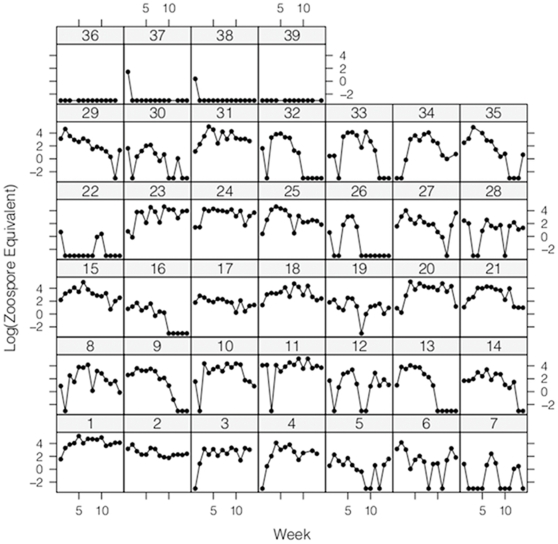
Lattice plot of individual *P. regilla* infection levels over 17 weeks. Infection levels are zoospore equivalents in log scale. The mortality threshold for *R. muscosa/sierrae* is 10^4^ zoospores, or 4 on the log scale. Numbers 1–35 are infected animals. Numbers 36–39 are control animals. Individual 1 was euthanized after the last week of the experiment.

Infections in laboratory *P. regilla* were variable but increased slightly over the 17 weeks of the study with a correlation of 0.437 for infection load over time using a Poisson distribution with individual as a random effect (P = 7.18e-8). Multiple individuals had infections consistently at or above 10^4^ zoospores, the level that causes death in *R. muscosa*/*sierrae* (Fig. 2) [18].

No animals died during the course of the study. Individual number 1 was euthanized one week after the end of the study due to lack of righting response or reaction to stimulus. This individual had a consistently high infection for almost five months before showing symptoms and had an infection level of 220,534 zoospore equivalents in the final week of the study. Of the 38 other infected frogs in the experiment, none showed symptoms associated with chytridiomycosis or any other disease during or after the study. Typical symptoms of chytridiomycosis are weight loss, lethargy, excessive skin shedding, muscle spasms, and loss of righting response or reaction to stimuli. Individuals gained weight during the course of the study in the control (r^2^ = 0.7492; P = 0.000031) and infected groups (r^2^ = 0.5202; P = 0.00241). Weights did not differ between the control and infected group in the first week (ANOVA P = 0.7066) or in the final week of the study (ANOVA P = 0.06916). Data from week four were omitted due to unreliable measurements because of low batteries in the scale used in weighing.

### Zoospore output

**Figure 3:**
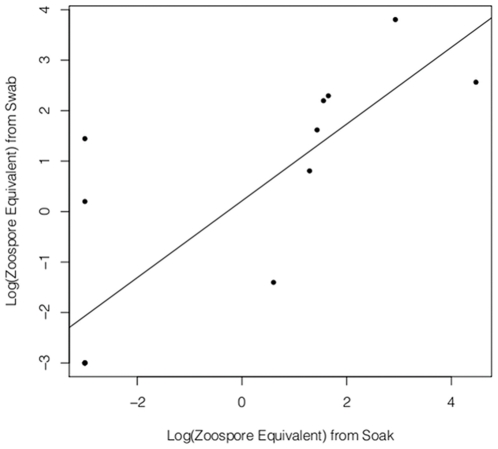
Correlation of *Bd* detection by swab versus soak. Soak and swab detection levels were correlated (r^2^ = 0.6543, P = 0.0002605, n = 10). Soaks were 77.8% effective at detecting zoospores detected by swab.

Seven of the fifteen soaks tested positive for *Bd* zoospores, and nine of the fifteen tested positive by skin swab. Thus the soak technique was 77.8% effective at detecting *Bd* DNA from soaks of infected animals. The zoospore equivalents detected by both methods were highly correlated (Fig. 3; r^2^ = 0.6543; P = 0.0002605). An average of 1,022 zoospore equivalents were detected in the soak water of each of the 9 infected animals for an average output rate of 68 zoospores per minute per individual.

### Histology

**Figure 4:**
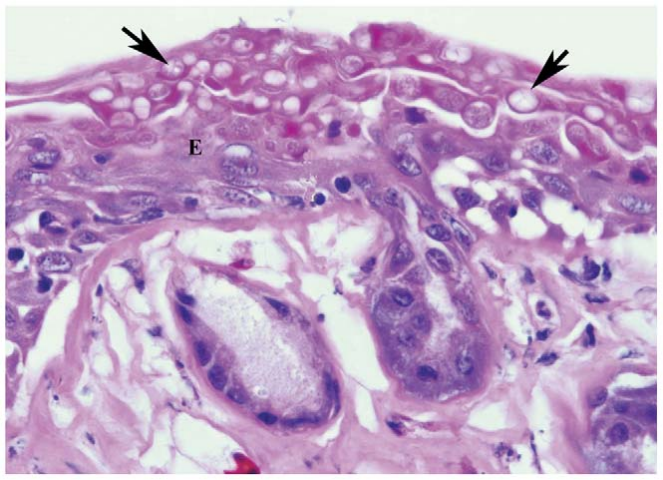
Histological view of skin from lethally infected individual. Photomicrograph of a histologic section of the ventral abdominal skin from *P. regilla* #1 (the only individual that died in captive experiment). There is diffuse severe hyperkeratosis with high numbers of *Bd* thalli (arrows). This lesion is consistent with lethal chytridiomycosis. E = epidermis.

Histologically observed skin lesions in a single animal (#1) that became ill and was euthanized soon after the end of the study consisted of moderate epidermal hyperplasia and hyperkeratosis with large numbers of fungal thalli consistent with *Bd* (Fig. 4). Lesions were diffuse meaning that they affected the entirety of ventral skin surfaces of the body, legs, and feet. The severity of the skin lesions in animal #1 was consistent with lethal chytridiomycosis as reported for highly susceptible amphibian species [19], [20], [21].

**Figure 5:**
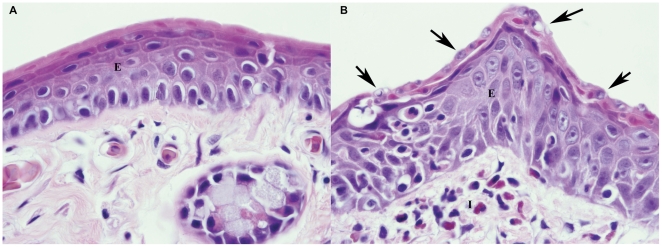
Histological views of skin from an infected but asymptomatic individual. Photomicrographs of histologic sections from the foot webbing of *P. regilla* # 32. In (a) the epidermis is well organized with minimal variation in nuclear size and a single keratinized layer consistent with normal foot skin. However, (b) shows an adjacent area of skin with disorganization of the epidermis (hyperplasia), hyperkeratosis and numerous *Bd* thalli (arrows). There are infiltrates of inflammatory cells in the epidermis and dermis. E = epidermis; I = inflammatory cells.

In10 apparently healthy animals that were euthanized, *Bd* thalli were observed in 4/5 animals with persistent, high intensity infections as determined by PCR (#5, 8, 27 and 32) and very small numbers of *Bd* thalli were observed in 1/5 animals (#24) with low intensity *Bd* infection. However, in contrast to the findings in animal # 1 with lethal chytridiomycosis, the lesions of epidermal hyperplasia and hyperkeratosis in apparently healthy animals were multifocal meaning that they occurred in discrete patches and adjacent areas of skin were notably free of *Bd* thalli with a normal underlying epidermis or had only very mild epidermal changes (Fig. 5). In the animals with high intensity infections either moderate numbers of *Bd* thalli were present and the degree of epidermal hyperplasia and hyperkeratosis was similar to a lethal *Bd* infection in a susceptible species (#8 and 32), moderate numbers of *Bd* thalli were observed in multiple locations on the skin (#27 and 32) or very low numbers of *Bd* thalli were observed (#5). In all animals with demonstrable *Bd* thalli there were mild to moderate infiltrates of inflammatory cells within the dermis and these occasionally extended into the epidermis. Inflammatory cells were predominantly granulocytes (neutrophils), but lymphocytes were observed in some areas. Lesions of mild epidermal hyperplasia and hyperkeratosis were also observed in most frogs regardless of infection intensity status and regardless of histologically observed *Bd* thalli.

## Discussion

Our results support the hypothesis that *P. regilla* may influence the spread and dynamics of *Bd* as a reservoir species based on the following evidence. *P. regilla* was found to be infected yet persisted in a basin from which *R. muscosa/sierrae* was almost entirely extirpated by a virulent chytridiomycosis outbreak. In the laboratory, *P. regilla* survived with extremely high loads of *Bd*. A patchy infection distribution may explain this tolerance and enhance the efficiency of *P. regilla* as a disease reservoir by allowing high infection intensity without symptoms or mortality. *P. regilla* emitted an average of 68 *Bd* zoospores per minute during the first 15 minutes of exposure to water, exhibiting high potential to affect the infectious zoospore pool to which other species and individuals are exposed.

The persistence of *P. regilla* in the field corroborates our laboratory findings, showing that when infected with *Bd* that is highly lethal to *R. muscosa/sierrae*, *P. regilla* did not experience population decline or range retraction. *R. muscosa/sierrae* experienced sustained infection from 2003 to the present raising the possibility that *Bd*-positive swabs collected from sympatric *P. regilla* may represent cross-species infection at the breeding pond and not sustained infection in the terrestrial phase of *P. regilla* when the opportunity to spread *Bd* across land would arise. Although our field sampling was conducted during the summer months of one year during the breeding season when *P. regilla* is most aquatic or in a fully aquatic larval stage, our laboratory infection monitoring suggests that *P. regilla* is capable of sustaining infection for long periods of time (at least four months) in the absence of constant contact with standing water. Further *Bd* sampling from wild *P. regilla* is necessary to further resolve the dynamics of *Bd* infection in the field in the species over time and in varying habitats.

In laboratory monitoring, adult *P. regilla* carried high levels of infection with only occasional morbidity or mortality, indicating that *P. regilla* would be capable of maintaining normal activity and dispersal patterns even when infected, potentially transporting *Bd* to new locations or harboring it long term in the environment as a result. In contrast, infected *R. muscosa/sierrae* in the lab usually experience an exponential increase in the number of zoospores until a threshold is reached (approximately 10^4^ zoospore equivalents) and the animal quickly dies [18], [22]. The differing disease outcomes in *R. muscosa/sierrae* and *P. regilla* correspond to what is seen in the field with rapid extirpation of *R. muscosa/sierrae* and the sympatric persistence of *P. regilla*. The ability to remain asymptomatic even with infection levels well above 10^4^ zoospores may make *P. regilla* a highly efficient reservoir in comparison to *X. laevis* and *R. catesbeiana*, which only carry low levels of infection as evaluated by histology [9], [10], [11].

Histology on apparently healthy *P. regilla* with high intensity *Bd* infection detected skin abnormalities similar to those observed with lethal *Bd* infection, but changes were limited to a patchy distribution which may explain the tolerance of *P. regilla* to *Bd*. The pattern of isolated patches of skin abnormalities could leave most infected *P. regilla* enough normal skin surface area to avoid electrolyte balance disturbances shown to cause death in amphibians with lethal *Bd* infection [23]. In contrast, the single *P. regilla* in this experiment that became sick (animal #1) had a diffuse heavy infection that would be expected to result in dysfunction of epidermal electrolyte transport and more closely resembles infection patterns seen in susceptible species like *R. muscosa/sierrae*. Interestingly, while the pattern of heavily infected skin patches may reduce mortality due to chytridiomycosis it also allows for high intensity *Bd* infections as measured by PCR that may lead to higher reservoir efficiency.

The reason for the patchy distribution of affected skin in *P. regilla* is unknown. Tolerance of *Bd* in other amphibian species may originate from innate immune activity of antimicrobial skin peptides or symbiotic bacteria [24], [25], [26], [27]. *R. muscosa*/*sierrae* populations that persist with chytridiomycosis have lower *Bd* prevalence and intensity and more *Bd*-inhibitory skin bacteria per host compared to more susceptible populations [28]. Since *P. regilla* in the lab had extremely high intensity infections, antimicrobial skin peptides and symbiotic bacteria do not appear to work in the same way to explain the ability of *P. regilla* to survive with chytridiomycosis, but may play a part in confining *Bd* infection to isolated patches of skin.

Similarly, it is unknown if adaptive host immune defenses could control the growth of *Bd* on the skin. Data suggest some species may mount adaptive immune responses including secretion into skin mucus of IgX, IgM, and IgY immunoglobulins that bind to *Bd* antigens [27], [29], [30]. However, it is unknown if these antibodies could confer protection against *Bd* infection in susceptible amphibian species and additional investigation is required. For instance, a single immunization trial in *R. muscosa* was ineffective in preventing infection and mortality [31]. It is possible that the inflammation observed in *P. regilla* from this study signifies underlying innate inflammatory or adaptive immune responses that help confine infection to patchy areas of skin. Significant inflammatory reactions in the skin are an inconsistent finding for chytridiomycosis in anurans, but have been reported in some salamanders that are more tolerant of chytridiomycosis [20], [32], [33].

The high *Bd* loads measured in *P. regilla* in the laboratory provide a challenge to current models predicting the outcome of *Bd* outbreaks [18], [22], [34]. The models presented in Briggs et al. 2010 show a density-dependent disease outcome based on individuals contributing zoospores to a common “zoospore pool” [34] Given our finding that *P. regilla* is capable of releasing an average of 68 zoospores per minute, the presence of *P. regilla* in a population will augment the *Bd* growth factor by effectively increasing the host population size. When these species overlap, the infection prevalence and intensity of *P. regilla* compounded with large population size may greatly affect the fate of the sympatric, *Bd*-susceptible *R. muscosa/sierrae* population. This emphasizes the necessity of including tolerant alternative hosts when predicting outcomes of *Bd* in any system. There are some limitations to the applicability of our soak results that should be considered for future studies. It is possible that our output estimate was inflated by a build up of *Bd* zoospores on the skin of the animal prior to submersion in soak water. It is necessary to do a time series of soaks to determine if the zoospore output rate changes over time. In addition, we did not test for viability of the zoospores present in the soak water, but only for the presence and quantity of *Bd* DNA. It would be valuable to use microscope observation or culture to determine what proportion of the zoospores is viable.

Our results elucidate the characteristics of an efficient *Bd* reservoir species and the same methods can be used to identify potential reservoir species in systems affected by chytridiomycosis around the world. When a new outbreak site is identified, identification of susceptible and reservoir species would help direct the allocation of conservation resources and result in more accurate prediction of local dynamics of the disease. Reintroduction of captive-bred animals from a susceptible species is likely to fail if reservoir species are present. Future studies must consider the full assemblage of host species in an area. Understanding the underlying physiological, environmental and immunological factors that lead to differences among species in susceptibility will lead to a better understanding of the patterns of disease susceptibility.

## Materials and Methods

### Habitat occupancy and field infection

Population presence or absence in water bodies in 60 Lakes Basin in Kings Canyon National Park was determined for *P. regilla* and *R. muscosa* with visual encounter surveys conducted between May and September every year from 2003 to 2010 (see Vredenburg et al. 2010). Skin swabs were collected in 2009 between June and August from 12 adult and larval *P. regilla*. Swabs were sterile pouched, plastic shaft, rayon- tip Dryswab™ (MWE MW113) stored in screw-top micro centrifuge tubes at 4°C immediately following swabbing. A standard swabbing protocol was used with 30 total strokes, 10 on each ventral side and upper thigh and 5 on the ventral side of each foot.

### Animal collection and husbandry

Thirty-nine adult *P. regilla* were collected between 1900 and 2300 h on April 9, 2010 from San Pablo Dam watershed (land managed by EBMUD, Orinda, CA, Contra Costa County). Six were gravid females and thirty-three were calling males. Thirty-five frogs were identified as infected with *Bd* by skin swab. Four naturally uninfected frogs served as controls. Frogs were housed individually on wet paper towels in plastic mouse cages with vented isolation tops in the animal care facility at San Francisco State University (Animal Use Protocol Reeder SFSU IACUC#A9-004). The room was kept between 16°C and 21°C and had a regular 12 h photoperiod. Cages were cleaned once a week and frogs were fed crickets.

### Weekly monitoring

Skin swabs for *Bd* PCR and weights were collected from each animal once a week over the 17-week experiment. Skin swabbing was conducted using the same protocol used in the field as described above. All frogs were weighed using a digital scale. Following the 17-week experimental period, frogs were continuously monitored for infection once a month.

### Zoospore output

To test for output of zoospores 15 frogs were removed from their housing containers and soaked for 15 minutes in 50 mL of filtered water. 50 µL of bovine serum albumen (BSA) were added to each water sample and they were stored at 4°C. The 50 mL of water were filtered through a 0.45 µm filter (Millipore Millex®-HA). A skin swab was taken immediately before soaking for comparison with the soak method. The success of the soak method at detecting *Bd* in infected animals was calculated by dividing the number of infections detected by soak by the number detected by swab. Infection intensities detected by each method were compared by correlation.

### Molecular methods

Swabs and soak filters were extracted using a PrepMan Ultra extraction and analyzed using a real-time quantitative PCR protocol described in Boyle et al. (2004) on an Applied Biosystems 7300 Real-Time PCR System. Negative extraction and PCR controls were run on each plate. Dr. Alex Hyatt (CSIRO AAHL Biosecurity Microscopy Facility, Australia) provided *Bd* standards. Zoospore equivalents per swab were calculated by multiplying the number of zoospores by 80 to account for dilution during the extraction and PCR processes.

### Histology

To determine the distribution of *Bd* infection and the degree of host response to infection histologic examination of skin sections was performed. Euthanasia was by immersion in tricaine methanesulfonate (MS222) buffered with NaHCO3 (Gentz 2007). One frog (animal #1) was euthanized after showing signs of illness and was frozen at −20°C for 8 months then thawed, fixed in 10% neutral buffered formalin and preserved in 70% ethanol. In addition, ten apparently healthy infected frogs from the experiment; five with high intensity and five with low intensity *Bd* infections as determined by PCR, were preserved in 10% neutral buffered formalin immediately after euthanasia. After fixation, frog carcasses were demineralized in hydrochloric acid (RDO Rapid Decalcifier, Apex Engineering Corp.) and cross-sections of the body (head at gular region and pelvic patch), hind legs (level of mid- femur and mid-tibiofibula) and feet (transverse section at approximately the level of the metatarsal phalangeal joints) were further processed for histologic examination. Tissues were automatically processed using a Tissue-Tek VIP vacuum infiltration processor (Sakura Finetek USA Inc.), embedded in paraffin and sectioned at 6 µm. Hematoxylin and eosin stained sections were examined by light microscopy by one of the authors (APP) blinded to infection status. Dorsal and ventral skin surfaces at each location were evaluated for the presence and distribution of *Bd* thalli. The presence of any skin lesions (host response) associated with *Bd* thalli such as epidermal hyperplasia, hyperkeratosis, and inflammatory cell infiltrates were noted. Features of epidermal hyperplasia include disorganization of keratinocytes, increased numbers of epidermal mitotic figures, and variation in epidermal nuclear size. Hyperkeratosis refers to increased numbers of keratinized epithelial cells on the outer surface of the epidermis (*stratum corneum*). For reference, the normal skin of *P. regilla* usually has a single layer of keratinized cells.

### Statistical analyses

All statistical analyses were performed using the statistical software R 2.11.0 [35] *Bd* zoospore infection levels were analyzed for trends over time using a generalized linear mixed model assuming a Poisson distribution to correct for non-normality on non-transformed zoospore equivalents with individual as a random factor. Mean weight gain or loss in the control and infected groups over time was analyzed using a linear regression model. Weights from the first and last weeks of the experiment were compared between the infected and control groups with a One-Way ANOVA to test for differences in weight over the course of the experiment. Results from soaks were compared to concurrent swab samples using a linear regression model to test the efficacy of the soak technique.
